# An initial evaluation of the safety of a disposable oscillating positive expiratory pressure device in patients with chronic obstructive pulmonary disease: a short-term pilot study

**DOI:** 10.1186/s12890-021-01689-y

**Published:** 2021-10-19

**Authors:** Kevin J. O’Sullivan, Valerie Power, Barry Linnane, Deirdre McGrath, Hilda Fogarty, Martina Ryan, Rebecca White, Conor Noonan, Eithne Mulloy, Leonard W. O’Sullivan, Colum P. Dunne

**Affiliations:** 1 Rapid Innovation Unit – Confirm Centre for Smart Manufacturing, School of Design and Health Research Institute, University of Limerick, Limerick, Ireland; 2 Centre for Interventions in Infection, Inflammation and Immunity (4i) and School of Medicine, University of Limerick, Limerick, Ireland; 3 University Hospital Limerick, Dooradoyle, Limerick, Ireland; 4 National Children’s Research Centre, Crumlin, Dublin 12, Ireland; 5 Paediatric Cystic Fibrosis Department, University Hospital Limerick (UHL), Limerick, Ireland; 6 St. John’s Hospital, Limerick, Ireland

**Keywords:** Chest physiotherapy, Lung function, Airway clearance therapy

## Abstract

**Background:**

Handheld oscillating positive expiratory pressure (OPEP) devices have been a mainstay of treatment for patients with hypersecretory conditions such as cystic fibrosis (CF) and chronic obstructive pulmonary disease (COPD) since the 1970s. Current devices are reusable and require regular cleaning and disinfection to prevent harbouring potentially pathogenic organisms. Adherence to cleaning regimens for respiratory devices is often poor and in response to this, a prototype disposable OPEP device—the ‘UL-OPEP’ (University of Limerick—Oscillating Positive Expiratory Pressure device)—was developed to mitigate the risk of contamination by pathogens. The device was previously evaluated successfully in a group of paediatric CF patients. The aim of the current study was to initially evaluate the safety of the prototype in patients with COPD over a period of 1 month to ensure no adverse events, negative impacts on lung function, exercise tolerance, or quality of life. Data on user experience of the device were also collected during post-study follow-up.

**Methods:**

A sample of 50 volunteer participants were recruited from pulmonary rehabilitation clinics within the local hospital network. The patients were clinically stable, productive, and not current or previous users of OPEP devices. Participants were invited to use a prototype disposable OPEP device daily for a period of 1 month. Pre- and post-study lung function was assessed with standard spirometry, and exercise tolerance with the 6-min-walk-test (6MWT). Quality of life was assessed using the St. George’s Respiratory Questionnaire (SGRQ), and user experience of the prototype device evaluated using a post-study questionnaire.

**Results:**

24 Participants completed the study: 9 were female. Overall median age was 67.5 years, range 53–85 years. Lung function, 6-min walk test, and SGRQ scores showed no significant change post-study. User feedback was positive overall.

**Conclusions:**

The results indicate that the UL-OPEP is safe to use in patients with COPD. No adverse events were recorded during the study or in the follow-up period of 2 weeks. The device did not negatively impact patients’ lung function, exercise tolerance, or quality of life during short term use (1 month), and usability feedback received was generally positive. Larger, longer duration studies will be required to evaluate efficacy.

*Registration* The study was approved as a Clinical Investigation by the Irish Health Products Regulatory Authority (CRN-2209025-CI0085).

## Background

Chronic obstructive pulmonary disease (COPD) is characterised by non-reversible airflow limitation due to an abnormal inflammatory response in the lungs. This response is caused by prolonged exposure to noxious particles or gasses, most commonly cigarette smoke, occupational dusts (from stone cutting, mining, metal grinding etc.) or other environmental dusts (e.g., woodworking) [1–3]. COPD is the fourth largest cause of death [4]. Blanco et al. estimated a global mean prevalence of 13.1% (95% CI) distributed as; Europe, 12.4% (8.8–16.0%); Africa, 13.9% (12.0–15.9%); America, 13.2% (10.5–15.9%); Asia, 13.5% (10.0–16.0%); and Oceania, 11.6% (9.8–13.1%) [5]. Patients are prone to exacerbations characterised by periods of acute worsening of symptoms such as dyspnoea, cough, airway inflammation, and excess sputum production (hypersecretion) [6–8]. Severe cases can require hospitalisation. Exacerbations are associated with lower quality of life, more rapid decline in lung function, and higher all-cause mortality [9–13]. Airway clearance and physiotherapy have been mainstays in treating hypersecretion since the 1950s [9], with the first description in the literature purporting the benefits published in 1901 [14]. Oscillating positive expiratory pressure (OPEP) devices are handheld, non-pharmacological adjuncts to chest physiotherapy. OPEP was designed to promote airway clearance by reducing the viscoelastic properties of the mucus [15–17], while splinting open collapsed airways [18] and increasing intrathoracic pressure distal to mucus plugging through collateral ventilation via the canals of Lambert and pores of Kohn [19]. OPEP therapy is achieved by blowing against a fixed or variable small-exit orifice which increases intrapulmonary pressure by limiting flow, introducing short increases in expiratory flow that act to mobilise secretions cephalad [19, 20]. The target range for mean intrapulmonary pressure during OPEP therapy is 10–20 cm H_2_O [19], with oscillations of at least 1 cm H_2_O from the mean. OPEP has been shown to be at least as effective as traditional chest physiotherapy for mobilising secretions [21, 22].

Although multiple handheld OPEP devices are available commercially at present, each current device requires periodic cleaning to prevent colonisation by potentially pathogenic organisms. Contamination of respiratory equipment is well documented either from exhalation into the device, or contamination of the mouthpiece [23–27]. This can be of particular importance to patients with already compromised pulmonary defences. The requirement to clean and disinfect these devices regularly can place a burden on patients and care givers, often leading to relatively poor compliance. Considering the COVID-19 pandemic, the cleaning and reuse of respiratory devices have been subject to increased attention in both home and healthcare facility settings [28, 29]. In response to potential contamination challenges arising from poor cleaning, our group designed a disposable OPEP device to eliminate this infection risk (the “UL-OPEP”), which has previously been studied in paediatric patients with CF [30].

The purpose of the current pilot study was to evaluate the initial safety of the UL-OPEP device for patients with COPD, as a first step in evaluating longer term efficacy. Safety was assessed as freedom from any device-related adverse events, no deterioration of lung function, exercise tolerance, or quality of life. Subjective user opinions of the device were captured in a post-study questionnaire.

## Methods

### Participants

Fifty participants were recruited at random from the COPD outpatient clinic lists in University Hospital Limerick (UHL), Ennis, Nenagh, and St. John’s Hospital Limerick, in the mid-west of Ireland. Inclusion criteria were that the patient be clinically stable at the time of recruitment, and that they were productive, and were not currently using an OPEP device. The exclusion criteria were any active exacerbations. The study duration of 1 month was based on a previously-registered short duration clinical trial of OPEP in COPD [31].

Potential participants were contacted via phone to invite them to participate and were then sent a written patient information leaflet, consent form, and clinic appointment by post to complete recruitment formally. Subsequently, they attended their appointments as scheduled across the four sites.

### Study

A single sample pre- and post-intervention pilot study design was employed, with all measurements and evaluations taking place in the outpatient clinic, except for the post-study questionnaire, which was administered by phone or email approximately 2 weeks after completion of the study protocol. At the first clinic visit, all participants were trained in the correct use of the UL-OPEP device by a chartered physiotherapist and provided with the following instructions for use:

You may use the *UL-OPEP* while sitting, standing, or lying down.

1. Inhale slightly more than usual, taking 1–2 s.
2. Place your lips on the *UL-OPEP* mouthpiece creating a firm seal. Without inflating your cheeks, exhale normally taking 5–6 s.
3. Repeat for 10–20 breaths, or as directed by your healthcare professional.
4. Suppress your desire to cough during these breaths. Remove the *UL-OPEP* from your mouth and exhale FORCEFULLY to help loosen the secretions to aid airway clearance, attempt 2–3 “huff” coughs to force the secretions from your airways.

Participants were instructed to use the UL-OPEP for 10 min per day as per the standard OPEP instructions across the local hospital network. Each participant was provided with a pack containing 30 single-day-use prototype disposable OPEP devices, instructions for use, and a patient information leaflet (PIL). The PIL contained contact information of the study Principal Investigators to contacted with any concerns during the study or to report any adverse events.

### Ethics

The study was conducted in compliance with the Good Clinical Practices protocol and the Declaration of Helsinki principle. Approval for this study was granted by the University Hospital Limerick Ethics Board (REC Ref: 020/18). The study was also approved as a Clinical Investigation by the Irish Health Products Regulatory Authority (CRN-2209025-CI0085). Informed consent was obtained from participants prior to commencement of the study. All data were codified anonymously with the ID key held separately in password protected files on an offline hard-drive in secure storage. All paper records were securely destroyed once tabulated into digital files.

### Instruments

**Fig. 1:**
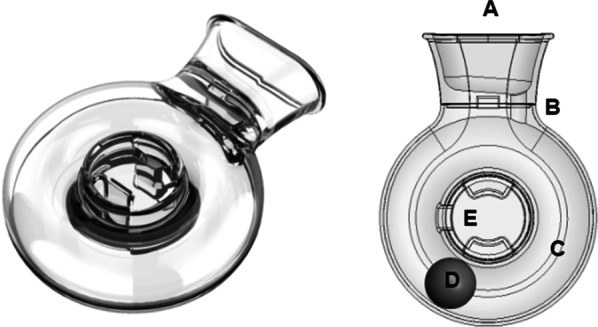
Left—A render of the prototype disposable UL-OPEP device, and, Right—mechanism of action [30]

The UL-OPEP prototype device (Fig. 1, Left) was used throughout the study. Figure 1 right shows the mechanism of action of the UL-OPEP as previously described by O’Sullivan et al. [30]. As the patient exhales into the mouthpiece of the device (A), the fixed orifice (B) restricts expiratory flow to generate a mean increase in intrapulmonary pressure. The expiratory flow exiting the orifice (B) into the circular track (C) causes the polymer ball (D) to revolve around the track. This causes the polymer ball (D) to periodically block the orifice (B), momentarily increasing intrapulmonary pressure to generate oscillations. The exhaled air escapes through the exhaust vent (E). The prototype device comprises two interlocking injection moulded shells manufactured from medical grade polycarbonate (Makrolon® 2858) by Synecco (Design, Development and Contract Manufacturing, Galway, Ireland). The Delrin ball was purchased from the Precision Plastic Ball Company Ltd. (Addingham, West Yorkshire, UK). Devices were assembled according to Good Manufacturing Practices (GMP) in a laminar flow hood in the University of Limerick before packaging and labelling. All details of the design, manufacture, and assembly of the device were submitted to the Health Products Regulatory Authority as part of the Clinical Investigation approval prior to commencement of the study (CRN-2209025-CI0085).

Lung function data were collected using the EasyOne Air Spirometer (NDD Medzintechnik AG, Switzerland) interpreted according to the Global Lung Function Initiative 2012 predicted values [32]. Routine lung function data collected comprised of force expiratory volume in one second (FEV_1_; litres and % predicted), forced vital capacity (FVC; litres and % predicted), and FEV_1_/FVC ratio. All spirometry assessments were performed by a chartered physiotherapist.

A 6-min walk test (6MWT) was performed pre- and post-study to assess exercise tolerance. Prior to attending the clinic appointment, participants were asked to wear comfortable clothing and shoes, to bring usual walking aids (canes, walkers etc.), to take all usual medication, and avoid any vigorous exercise. The test was completed on a flat straight corridor, 30 m long, marked every 3 m. The test facilitator tracked elapsed time on a stopwatch, and recorded distance (laps or part thereof) on a flow sheet. Verbal instructions were issued to participants to walk as far as possible during the 6 min without jogging or running. Participants were permitted to slow down, stop, and rest as required, but asked to continue walking again when able. During the test, SpO_2_ was recorded on a wrist mounted pulse oximeter (Wrist0x_2_ Model 3150, Nonin Medical Inc., Plymouth, MN, USA) and an average was generated for the duration. The test was terminated early if the participant exhibited chest pain, intolerable dyspnoea, abnormal gait, HR > Predicted max (220-age), persistent Sp02 < 85%, severe physical or verbal fatigue, pale or ashen appearance, or diaphoresis.

Disease-specific impacts on participants’ health and quality of life were assessed using the St. George’s Respiratory Questionnaire (SGRQ) [33] desktop app (developed by Copenhagen Clinical Research Development, Marco Gelpi MD, Jonathan Argentiero, and Andreas Ronit MD). The SGQR has been validated for use in COPD, asthma, and a revised version for use in CF (SGRQ-R). The questionnaire comprises 3 components; symptoms (quantifying stress due to respiratory symptoms), activity (effect of disturbances to mobility and physical activity), and impact (psychosocial impact of respiratory disease). The total score (0–100) is reflective of the global estimate of respiratory health, with a higher score indicating poorer health.

Two weeks post completion of the study, participants were followed-up by phone or email and a post-study questionnaire was administered to collate data on users’ experiences and opinions of the prototype device using 5-point Likert scales (Strongly Agree to Strongly Disagree).

### Analysis

All data were initially collated, codified and graphed in Microsoft Excel. Statistical analysis was completed using SPSS Version 28 (IBM, New York, USA). Shapiro–Wilks tests were used to assess normality of the data, and Students’ paired samples t-tests were used to evaluate differences pre- and post-study results. A two-sided significance was set at 0.05.

## Results

Of the 50 participants recruited a total of 24 completed the study; of whom 9 were female, overall median age 67.5 years, range 53–85 years. Twenty-three participants withdrew or failed to attend the follow-up evaluation and a further 3 participants were excluded from analysis as they were found to have been using an OPEP device prior to commencing the study. No device-related adverse events were reported during the study or in the follow-up period.

In total, there were 24 usable data sets for spirometry, 18 usable sets for the 6MWT, 20 usable data sets for the SGRQ and 24 responses to the post-study questionnaire.

Shapiro–Wilks tests showed no significant departure from normality in any of the baseline data, with all *p* values > 0.05. Specifically, spirometry results were normally distributed at baseline for FEV_1_ (Litres) W(24) = 0.920, *p* = 0.058; FVC (Litres) W(24) = 0.974, *p* = 0.767; and FEV_1_/FVC Ratio W(24) = 0.713, *p* = 0.713. A summary of descriptive statistics for the participants at baseline and follow-up is shown in Table 1.

**Table 1:** Descriptive statistics at baseline and follow-up for spirometry, 6MWT, and SGRQ results

	Spirometry			6MWT		SGRQ			
	FEV_1_ (L)	FVC (L)	FEV_1_/FVC	Distance (m)	Mean SpO_2_ (%)	Symptoms	Activity	Impact	Total
*Baseline*									
(n)	24	24	24	18	18	20	20	20	20
Mean	1.410	2.744	0.514	404.72	93.05	60.63	56.14	27.96	42.07
95% CI Lower	1.129	2.383	0.442	345.32	91.54	53.03	43.9	21.46	34.76
95% CI Upper	1.692	3.105	0.587	464.11	94.56	68.23	68.38	34.45	49.37
Median	1.275	2.750	0.508	422.00	92.50	60.92	52.84	28.86	40.01
Standard Dev	0.666	0.855	0.173	119.43	3.03	16.23	26.14	13.87	15.6
Min	0.550	1.180	0.230	180.00	85.00	21.65	7.64	6.04	16.08
Max	2.760	4.390	0.840	575.00	99.00	90.73	100	55.67	70.63
Skewness	0.642	0.180	0.019	− 0.439	− 0.526	− 0.293	0.098	0.279	0.078
Kurtosis	− 0.646	− 0.570	− 0.915	− 0.786	2.217	0.73	− 0.931	− 0.505	− 0.934
*Follow up*									
(n)	24	24	24	18	18	19*	20	20	20
Mean	1.460	2.764	0.521	431.28	92.84	60.99	58.74	31.82	42.8
95% CI lower	1.147	2.400	0.446	372.14	91.39	51.45	48.89	23.66	36.71
95% CI upper	1.774	3.128	0.597	490.40	94.29	70.52	68.58	39.99	48.89
Median	1.280	2.700	0.547	435.00	93.17	5915	60.335	30.94	39.6
Standard Dev	0.742	0.861	0.179	118.90	2.91	19.78	21.03	17.44	13.01
Min	0.560	1.170	0.220	270.00	86.00	32.26	22.2	4.76	14.04
Max	3.290	4.840	0.880	655.00	97.00	97.05	92.45	79.14	65.12
Skewness	0.851	0.415	0.600	0.276	− 1.038	0.096	− 0.344	0.911	0.018
Kurtosis	0.206	0.154	− 0.784	− 1.003	0.994	− 0.975	− 0.924	1.69	− 0.131

^*^The Symptom score for participant ID66 was not exported by the SGRQ app

**Fig. 2:**
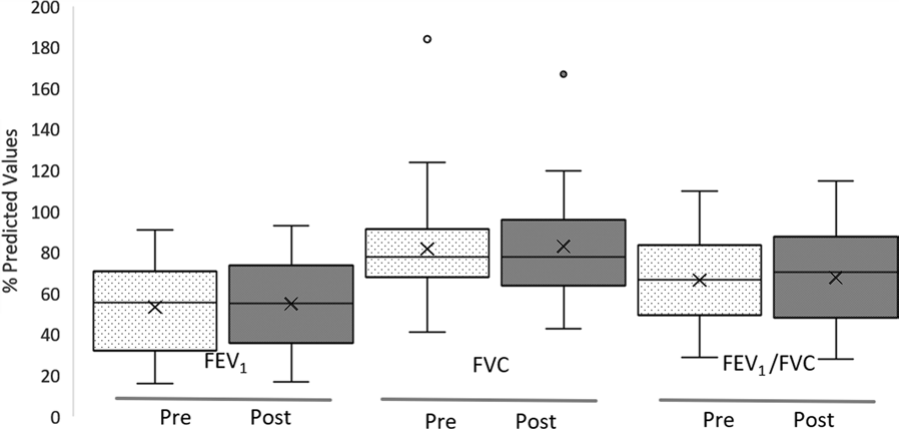
Pre- and post-study values for FEV1, FVC (% predicted), and FEV1/FVC Ratio Box-whisker plot shows mean (x), median (horizontal line), interquartile range (box), and minimum/maximum (whiskers)

There were no significant difference in participants scores for FEV_1_ (Litres) pre- (${\overline{\text{x}}}$ = 1.41, SD = 0.66) and post- (${\overline{\text{x}}}$ = 1.46, SD = 0.74) study; t(23) = − 1.440, *p* = 0.163 or, FVC (Litres) pre- (${\overline{\text{x}}}$ = 2.74, SD = 0.86) and post- (${\overline{\text{x}}}$ = 2.76, SD = 0.86) study; t(23) = − 0.284, *p* = 0.779 or, FEV_1_/FVC Ratio pre- (${\overline{\text{x}}}$ = 0.51, SD = 0.17) and post- (${\overline{\text{x}}}$ = 0.52, SD = 0.17) study; t(23) = − 0.881, *p* = 0.387 (Fig. 2).

**Fig. 3:**
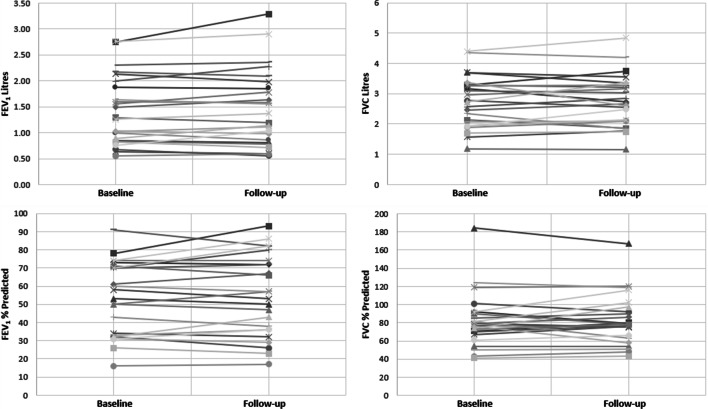
Line plots of individual change between baseline and follow-up for FEV_1_ Litres (top left), FEV_1_% Predicted (bottom left), FVC Litres (top right), and FVC % Predicted (bottom right)

The line plots shown in Fig. 3 represent the variability in changes between baseline and follow-up for each of the individual participants (n = 24) for FEV_1_ Litres, FEV_1_% Predicted, FVC Litres, and FVC % Predicted.

**Fig. 4:**
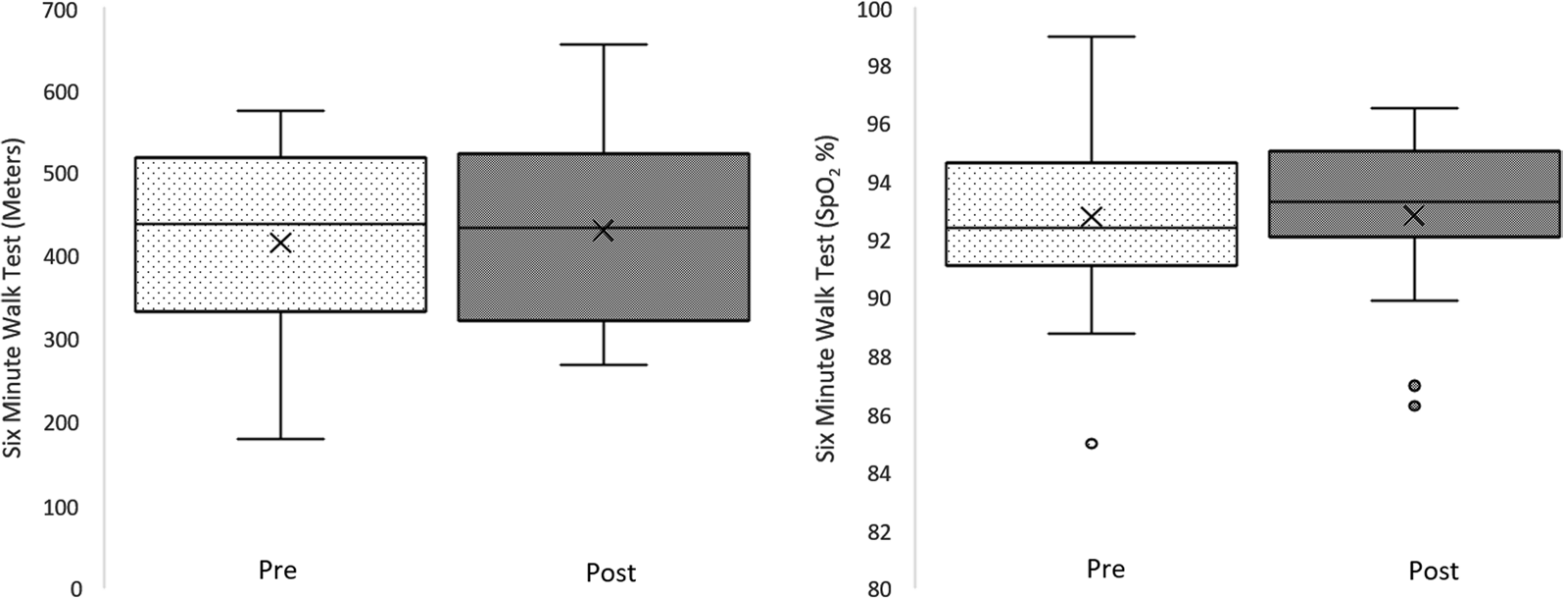
Six Minute Walk Test results—Meters (Left), and SpO_2_ (Right) pre- and post-study. Box–Whisker plot shows mean (x), median (horizontal line), interquartile range (box), and minimum/maximum (whiskers)

**Fig. 5:**
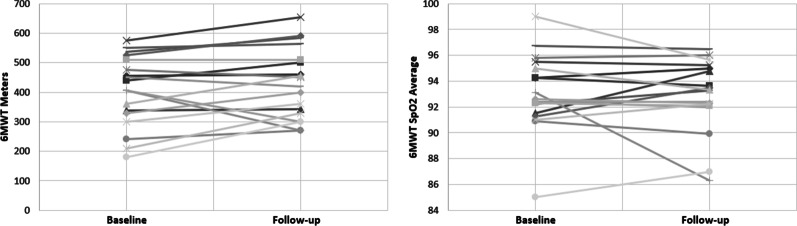
Line plots of individual change between baseline and follow-up for 6MWT distance (left) and average SpO_2_ (right)

The results for 6MWT (n = 18) showed no statistical difference in distance (meters) pre- (${\overline{\text{x}}}$ = 404.72, SD = 119.43) and post- (${\overline{\text{x}}}$ = 431.27, SD = 118.90) study; t(17) = − 1.618, *p* = 0.124. Nor was there any difference in average SpO_2_ during the 6MWT pre- (${\overline{\text{x}}}$ = 93.05, SD = 3.03) and post- (${\overline{\text{x}}}$ = 92.84, SD = 2.91) study; t(17) = 0.390, *p* = 0.702 (Fig. 4). Individual results for distance and average SpO_2_ are shown in Fig. 5.

**Fig. 6:**
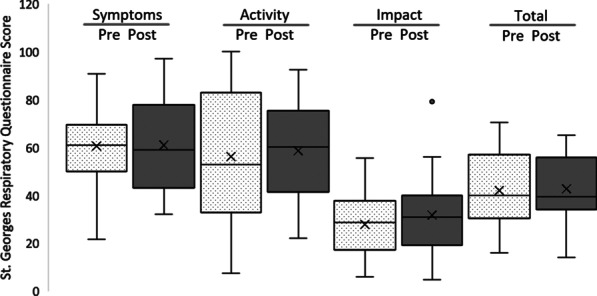
SGRQ scores pre- and post-study. Box–Whisker plot shows mean (x), median (horizontal line), interquartile range (box), and minimum/maximum (whiskers)

**Fig. 7:**
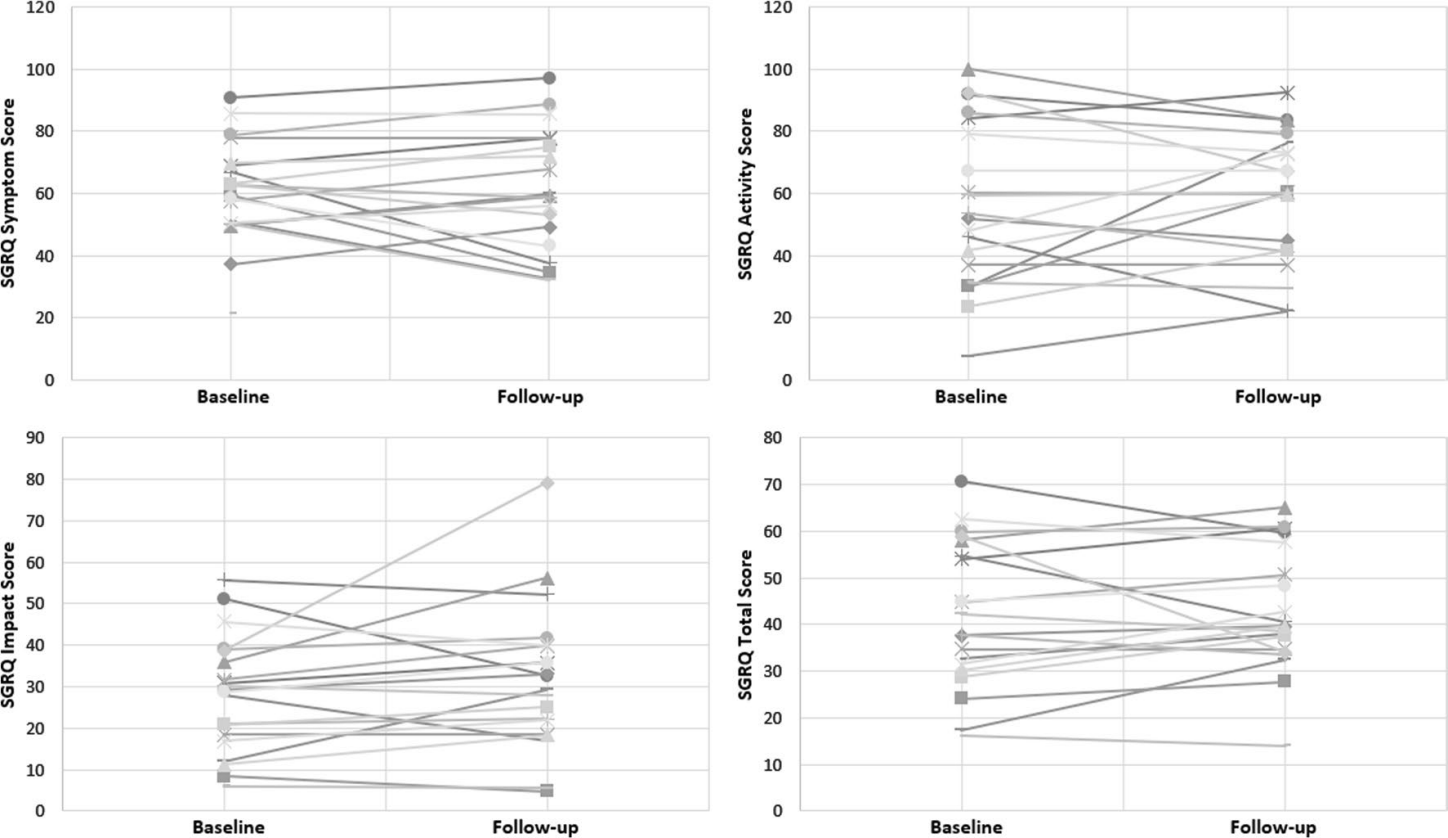
Line plots of individual change between baseline and follow-up for the SGRQ results—symptoms (top left), activity (top right), impact (bottom left), and total score (bottom right)

The participant scores at baseline and follow-up for the St. George’s Respiratory Questionnaire (n = 20) are shown in Fig. 6. No significant differences were observed in scores pre- and post-study across the three dimensions of symptoms, activity, and impact (paired sample t-tests, all *p* > 0.05), or the total score pre- (${\overline{\text{x}}}$ = 42.07, SD = 15.60) and post- (${\overline{\text{x}}}$ = 42.80, SD = 13.01) study; t(19) = − 0.347, *p* = 0.733. Individual results from baseline to follow-up are shown for the four SGRQ dimensions in Fig. 7.

**Fig. 8:**
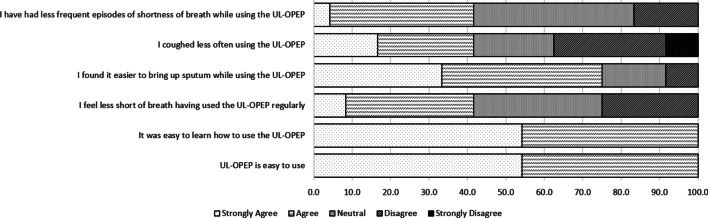
User experience of the prototype disposable UL-OPEP device

In the post-study questionnaire, the majority of participants reported that the prototype device: was easy to use (54.2% Strongly Agree; 45.8% Agree), and was easy to learn how to use (54.2% Strongly Agree; 45.8% Agree), while 41.6% felt less short of breath having used the prototype regularly (8.3% Strongly Agree; 33.3% Agree; 33.3% Neutral, 8.3% Disagree). Most felt that it was easier to bring up sputum while using the device compared with no OPEP use (33.3% Strongly Agree; 41.7% Agree; 16.7% Neutral, 8.3% Disagree) (Fig. 8).

In total, 45.8% (n = 11) of participants reported an increase in the amount of expectorated sputum, albeit that 16.6% (n = 4) reported a reduction, and 37.5% (n = 9) reporting no change.

## Discussion

The current study evaluated initial safety of the UL-OPEP device for patients with productive COPD, as a first step towards evaluating longer term efficacy. The pilot study was performed with this patient group as previous evaluations of the UL-OPEP device were undertaken exclusively with paediatric patients with CF [30], whom the device was originally designed for. The study met its primary objectives, in that the device appears safe to use, with no deterioration in lung function as assessed via spirometry, no decline in exercise tolerance, no adverse events observed or reported during the study or follow-up period, and no deterioration of participants’ general health and wellbeing. While it is acknowledged that this was a short duration pilot study, participants who completed the study expressed a generally favourable view of the prototype device. Those participants who were more productive found the device beneficial in expectorating more sputum than before.

While there were no statistically significant changes in any of the variables evaluated, the individual participant changes between baseline and follow-up demonstrate the broad variability expected in such a heterogeneous population. Of the 24 participants who completed our study, six declined or were unable to complete the pre- or post-intervention 6MWT (due to unacceptably low SpO_2_ or shortness of breath), while four participants failed to complete the pre- or post-study SGRQ due to a stated ‘lack of time’. Three participants were excluded from the analysis when it was established they had been using an OPEP device prior to enrolment in the study. The enrolment of these participants was attributed to confusion by the participants as to whether they had used the *UL-OPEP* before, to which they naturally responded in the negative.

Unfortunately, there was a high dropout rate from the study, with 46% of the participants either withdrawing from the study early or failing to attend the post-study assessment. Unlike results previously reported by the authors relating to paediatric CF settings, adherence to and completion of the protocol in this study were poor. Those participants that failed to attend the post-study assessment declined to complete the post-study questionnaire when contacted for follow-up, and no explicit reason was given. Poor adherence to treatment regimens is well documented in patients with respiratory conditions [34, 35]. Sahin [36] found that of 359 COPD patients referred to a pulmonary rehabilitation program, 41% failed to complete the program (which is broadly in line with the rate seen in our study)—a range of sociodemographic, behavioural, psychological and physiological characteristics were identified as contributors in the group that failed to complete the Sahin study.

As participants in the present study were not previous users of OPEP devices prior to commencing this study, the absence of OPEP therapy from participants’ long-term treatment for a condition with which they have lived for some time may have negatively impacted on the perceived effectiveness of OPEP therapy in general, and participants’ motivation to use the UL-OPEP device. Some drop-out from the study may also be explained, in part, by participants being disheartened when no significant improvements in their condition were immediately evident. In that context, the lack of motivation in these patients and associated low adherence to prescribed practices lend themselves to the potential benefit of a disposable offering. Even a modest schedule of replacing an OPEP device once a month could represent a considerable increase in frequency for some patients who may rarely, if ever, clean their reusable devices despite available evidence that they may act as reservoirs for potentially pathogenic organisms [27, 37].

While there are several small studies reporting the benefits of OPEP in reducing exacerbations in COPD, there is limited but growing evidence from large clinical trials and real-world studies [9, 38]. Burudpakdee et al. [39] reported that use of the Aerobika OPEP device (Trudell Medical, Ontario, Canada) reduced exacerbations by 30% compared to no PEP/OPEP after an inpatient stay in a sample of 405 patients, while Tse et al. [9] found that the use of OPEP significantly reduced all cause inpatient admissions at 12 month follow up in a retrospective study of 2476 patients. A recent systematic review of the use of OPEP to augment sputum clearance in COPD by Alghadmi et al. [40] found that while OPEP devices can have a positive impact in COPD, confidence in the effect size is low, and the quality of evidence was low to moderate grade overall.

Of the modest number of published randomised trials evaluating OPEP in COPD, the clinical presentation is described simply as either ‘acute’ or ‘stable’ COPD [38]. This method of characterisation, while employed in this study, is inadequate in practice to describe the clinical phenotype in such a varied condition. No information could be discerned regarding the amount of sputum produced by any control or intervention groups pre- or post-study. This is an important but overlooked element of patient selection for evaluation of the clinical efficacy of OPEP. Indeed, while sputum production is a symptom of great concern for patients, the Global Initiative for Obstructive Lung Disease 2019 and European Respiratory Society/American Thoracic Society guidelines make no mention of sputum clearance [8, 41]. This ambiguity in stratifying patients is further compounded by the advancing age of most COPD cohorts (the average in this study was 68.6 years). There is little consideration given to age-related changes such as increased multimorbidity, polypharmacy, severe deconditioning of pulmonary tissue, and so called ‘senile’ emphysema, separate to hypersecretion [42].

A recurrent theme in the literature surrounding OPEP use is the impact of non-clinical factors in the selection of airway clearance techniques and devices [19, 34, 43]. The importance of patient preference as a factor in the efficacy of the OPEP devices should not be underestimated. The effectiveness of any airway clearance technique is influenced strongly by a patient’s satisfaction, motivation, and the perceived effectiveness of the technique [44, 45]. The best OPEP device for a patient may be *whichever one they will use*.

Future studies evaluating the use of the UL-OPEP in the treatment of COPD would benefit from more careful screening of potential participants, concentrating on those who are more productive and who may benefit most from OPEP therapy [17, 46], and those who are known to be more adherent to treatment regimens in general.

### Limitations

The relatively small sample size limits the generalisation of these results to the wider COPD population; a larger longer duration study will be required to evaluate the long-term clinical efficacy of the device. This pilot study design was based on previous brief duration evaluations [31], however studies of 6–13 months are employed more commonly when evaluating OPEP in COPD [9]. This was impractical for the purpose of the current pilot study, which sought only to assess the initial safety of the device and to collect usability data on the design.

The authors acknowledge that the use of a daily disposable device for patients with COPD would represent a significant increase in annual cost compared to current commercially available devices. However, replacing the disposable UL-OPEP device less frequently (e.g., weekly or monthly) would not only be comparable or lower than current device costs, but may have the benefit of reducing the costs associated with additional treatment of infection due to insufficient cleaning practices. While the costs of a single exacerbation are difficult to quantify, due to variability across geographic location and public/private healthcare systems, the average cost per severe COPD exacerbation in one university hospital in Greece was reported as €1,711 [47], while the annual per patient direct costs has been estimated at up to €10,701 in Norway [48].

Separate to cost, the frequency of replacing a disposable respiratory device is cause for genuine reflection in the context of environmental impact and the current global climate crisis. Petroleum based polymers are of huge importance to the medical-tech sector [49] and are used extensively in the production of medical devices [50], which are often single use or regularly changed and disposed of. Indeed, 90% of medical device waste is generated by single use disposables, which are employed increasingly over reusable devices to avoid the risk of contamination. About 15% of medical waste produced daily is classified as hazardous and incinerated, with the remaining 85% destined for landfill [51]. The benefits of reduced risk associated with disposables will undoubtedly, therefore, be reliant on the rapidly developing field of biopolymers.

## Conclusions

The pilot study reported no device-related adverse events and no deterioration in lung function arising from use of the prototype UL-OPEP device, albeit over a short duration of 1 month. Exercise tolerance and quality of life were maintained throughout the study. The prototype device was considered easy to learn and use by participants, with self-reported expectoration of mucus improved in 46.8% of those who completed the study.

In the current coronavirus pandemic, the rationale for a disposable alternative to currently available OPEP devices is compelling particularly for COPD patients who face the daunting prospect of inpatient stays during an exacerbation. However, the environmental impact of disposable polymer devices is an ever-increasing cause for concern. It is also reasonable to speculate that the cost of routine or long-term daily use may limit accessibility unless supported by local health care systems or health insurance, and that less frequent replacement may be more economically viable, while still retaining the benefits of infection reduction.

Despite these concerns, the potential benefits of employing a cost-effective disposable OPEP device for short term specific-purpose use in patients with COPD such as travel, clinic evaluations, and in-patient admissions for exacerbations may be useful.

## Data Availability

The datasets used and/or analysed during the current study are available from the corresponding author on reasonable request.
